# Dry-heat inactivation of “*Mycobacterium canettii*”

**DOI:** 10.1186/s13104-017-2522-z

**Published:** 2017-06-09

**Authors:** Djaltou Aboubaker Osman, Eric Garnotel, Michel Drancourt

**Affiliations:** 1 Aix Marseille Université, URMITE, UMR CNRS 7278, IRD 198, INSERM 1095. IHU Méditerranée Infection, 13005 Marseille, France; 2 Institut de Recherche Médicinale (IRM), Centre d’Études et de Recherche de Djibouti (CERD), Djibouti, Djibouti; 3 Hopital d’Instruction des Armées LAVERAN, 34 Boulevard Laveran, 13384 Marseille, France

**Keywords:** “*Mycobacterium canettii”*, Smooth tubercle bacillus, Thermal tolerance, Environmental reservoir

## Abstract

**Objective:**

“*Mycobacterium canettii*” is responsible for non-transmissible lymph node and pulmonary tuberculosis in persons exposed in the Horn of Africa. In the absence of direct human transmission, contaminated water and foodstuffs could be sources of contamination. We investigated the dry-heat inactivation of “*M. canettii”* alone and mixed into mock-infected foodstuffs by inoculating agar cylinders and milk with 10^4^ colony-forming units of “*M. canettii*” CIPT140010059 and two “*M. canettii*” clinical strains with *Mycobacterium tuberculosis* H37Rv as a control.

**Results:**

Exposed to 35 °C, *M. tuberculosis* H37Rv*, “M canettii”* CIPT140010059 and *“M. canettii”* 157 exhibited a survival rate of 108, 95 and 81%, which is significantly higher than that of *“M. canettii”* 173. However, all tested mycobacteria tolerated a 90-min exposure at 45 °C. In the foodstuff models set at 70 °C, no growing mycobacteria were visualized. This study supports the premise that “*M. canettii*” may survive up to 45 °C; and suggests that contaminated raw drinks and foodstuffs but not cooked ones may be sources of infection for populations.

## Background

*“Mycobacterium canettii”,* a smooth-looking colony and variant of the tuberculosis agent *Mycobacterium tuberculosis,* is regularly isolated in patients diagnosed with lymph node and pulmonary tuberculosis and exposed to the Republic of Djibouti and neighboring countries in the Horn of Africa [1, 2]. “*M. canettii”* is characterized by a rapidly growing tuberculous mycobacterium which produces glossy and shiny colonies [1]. The generation time of the *‘M. canettii’* So93 strain, the first well-characterized isolate, was found to be of 17 h, which is significantly shorter than the generation time measured in parallel for *M. tuberculosis* and *Mycobacterium bovis* [1]. This aspect was confirmed by growing five representative strains in both liquid and solid media [3]. Since no inter-human transmission has been observed for “*M. canettii”*, a yet unknown environmental reservoir has been suggested [2, 4]. Clinical data obtained along with a few experimental data suggest contaminated drinking water, beverages and foods as potential sources of contamination [5]. This hypothesis raises the question of whether “*M. canettii”* could be inactivated by dry heat, a common process to inactivate foodborne pathogens and prevent foodborne diseases [6, 7]. No data are available regarding this question. We therefore studied the thermal tolerance of “*M. canettii”* and we set up a protocol to measure the viability of the mycobacteria subjected to a range of temperatures and exposure times.

## Main text

### Bacterial strains

The reference “*M. canettii”* CIPT140010059 strain and the reference *M. tuberculosis* H37Rv strain were acquired from the Collection de l’Institut Pasteur, Paris, France. Two “*M. canettii”* clinical isolates (“*M. canettii*” 157 and “*M. canettii*” 173) were used in this study. *M. canettii* 157 and 173 strains were isolated from patients with pulmonary and lymph node tuberculosis, living in Djibouti for 3 and 5 years before clinical symptoms, respectively. The patient with lymph node tuberculosis was co-infected by HIV. Clinical strains were stored at −80 °C and frozen in glycerol until used. Partial sequencing of the *ropB* and *gyrB* genes performed as previously described [8, 9] was used to confirm identification of the isolates. The mycobacteria were cultured in Middlebrook 7H9 broth (Becton–Dickinson, Le Pont-de-Claix, France) enriched with oleic acid-albumin-dextrose-catalase (OADC) (Becton–Dickinson) for 2 weeks for *M. tuberculosis* H37Rv and 6 days for the *“M. canettii”* mycobacteria at 37 °C in a 5% CO_2_ atmosphere. Mycobacteria were suspended at a final concentration of 10^5^ mycobacteria/mL in sterile phosphate buffered saline (PBS). The inoculum was calibrated with a turbidimeter (Biolog, Hayward CA, USA) at 590 nm. To avoid cell clumping, the mycobacteria were dispersed by expelling the suspension seven times through a sterile 26**-**gauge needle attached to a 1-mL syringe (Becton–Dickinson). The dispersion of mycobacteria was confirmed by microscopic observation after Ziehl–Neelsen staining.

### Heat treatment of isolates

A 0.1-mL volume of mycobacterial suspension was mixed with 0.9 mL of Middlebrook 7H9 broth supplemented with OADC at room temperature. Each microtube was immersed in a dry heating block system (Grant Instruments Cambridge Ltd, England) at temperatures ranging from 25 to 75 °C with an incrementation of 10°. Four different durations of exposure (15, 30, 60 and 90 min) were tested for each temperature. After heating, the vials were removed and 0.1 mL of heated suspension was immediately seeded on Middlebrook 7H10 agar (Becton–Dickinson) supplemented with OADC growth supplement and the rest was kept at 4 °C. The experiment was carried out to determine the survival curve for each isolate.

The presence of viable mycobacteria was determined by mycobacterium colony counting on Middlebrook 7H10 agar after two serial dilutions and incubation for four and 3 weeks respectively for *M. tuberculosis* H37Rv and “*M. canettii”* strains at 37 °C in a 5% CO_2_ atmosphere. Reproducibility was evaluated in two heat treatment assays. Colony identification was confirmed by Ziehl–Neelsen staining and matrix-assisted laser desorption/ionization time-of-flight mass spectrometry identification [10]. As a control, 1 mL of non-inoculated Middlebrook 7H9 broth supplemented with OADC growth supplement was treated according to the same procedure.

### Foodstuff models

To study the thermal inactivation of mycobacteria, we used an agar cylinder [11] and milk as a food model [12]. Agar cylinders were prepared and inoculated by using a 50-mL syringe (Becton–Dickinson). A 150-mL volume of molten Middlebrook 7H10 agar (Becton–Dickinson) at 50 °C was inoculated with 1.5 mL of a 10^4^ mycobacteria/mL suspension and 50 mL of this mixture were quickly poured into the syringe. Thermal inactivation experiments were performed with the agar cylinders prepared as above. The syringes were heated at different times of exposure (15, 30, 60 and 90 min) by submerging them in a circulating water bath (Memmert GmbH, Schwabach, Germany) set at 70 °C. The syringe was kept at room temperature to allow the agar to solidify in order to be cut in uniform slides. After heat treatment, the cylinders were cut into lengths of 2–3 cm and the sections were incubated at 37 °C in a 5% CO_2_ atmosphere for the same periods as described above. All thermal inactivation experiments were duplicated. A MZ-FLIII fluorescence microscope (Leica, Nanterre, France) equipped with a green fluorescent protein filter and an ICA digital camera (Leica) was used to detect autofluorescent mycobacterial colonies [13, 14]. Then, a 0.9-mL volume of pasteurized milk was inoculated with 0.1 mL of a 10^4^ mycobacteria/mL suspension, vortexed and heated in a dry bath (Grant Instruments) at 70 °C at different times of exposure (15, 30, 60 and 90 min). A 0.1-mL volume was immediately seeded on Middlebrook 7H10 agar (Becton–Dickinson) supplemented with OADC and the rest was kept at 4 °C at the end of each heat treatment. Viable colonies were numerated as above. Non-inoculated Middlebrook 7H10 agar and milk were used as negative controls.

### Statistical analysis

All experiments were replicated twice. The data were analyzed with ANOVA using publicly available software (UPMC, Paris, France) to determine if there were significant differences (p < 0.05) in the mean values of colony-forming units (CFU).

### Results

**Fig. 1:**
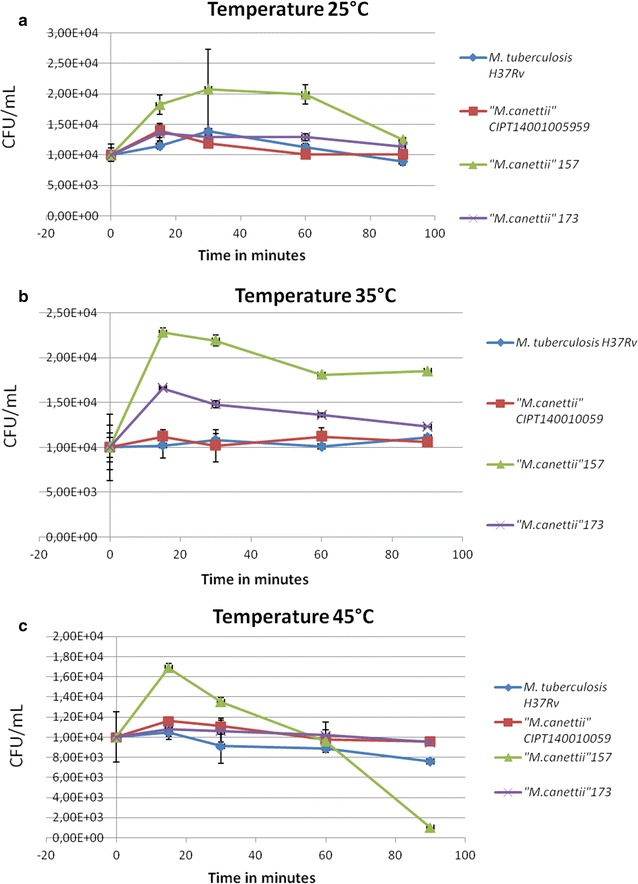
Survival curves for *M. tuberculosis* H37Rv, “*M. canettii*” CIPT140010059, “*M. canettii*” 157 “*M. canettii*” 173 **a** 25 °C, **b** 35 °C, **c** 45 °C

The negative controls examined in the study remained negative since no colony was observed either with the naked eye or by microscopy. After a 75-min exposure to 25 °C the mean number of colonies for “*M. canettii*” 157 showed a greater decrease (from 1.82 × 10^4^ ± 200 colonies at 15 min to 1.25 × 10^4^ ± 500 colonies at 90 min) than for the three other mycobacteria under assay (p = 3.179^−6^). The same observation holds true for the 75-min exposure to 35 and 45 °C (Fig. 1). Indeed, a 75-min exposure to 45 °C killed *“M. canettii”* 157 but not the other mycobacteria under assay (Fig. 1). Further comparison of the mean number of colonies for each strain after a same exposure at 25, 35 and 45 °C indicated a significant reduction for the two *“M. canettii”* clinical isolates (*“M. canettii”* 157 and *“M. canettii”* 173) compared to the reference *M. tuberculosis* H37Rv and *“M. canettii”* CIPT140010059 (p = 0.035 and p = 0.004). Regardless of the exposure time, no colonies were observed for any of the four mycobacteria after exposure to 55 °C which represents the highest temperature tolerated by the *M. tuberculosis* complex mycobacteria tested in this study. The counting of viable mycobacteria indicated that two of three tested *“M. canettii”* isolates tolerated 25 to 45 °C after a 90-min exposure but were all inactivated at temperatures ≥55 °C and exposure time ≥15 min.

Moreover, the foodstuff models set at 70 °C yielded no growing mycobacteria for any of the four investigated mycobacteria.

### Discussion

The purpose of this study was to evaluate the thermal inactivation of “*M. canettii”* for which a yet unknown reservoir in the environment is suspected [2, 4]. Clinical data indicate the digestive tract as an alternative route of contamination for *“M. canettii”* [5]. Dry heating is a well-recognized method of inactivation of foodborne pathogens [15]. There are a few examples of food-borne mycobacteria including *Mycobacterium avium* subsp. *paratuberculosis* found in dairy, beef, cattle and sheep and considered as an emerging foodborne pathogen and the etiologic agent of Johne’s disease [16]. This pathogen has been shown to be heat-inactivated [17]. As for tuberculosis, *Mycobacterium bovis* is transmissible by the consumption of unpasteurized milk and responsible for extra-pulmonary tuberculosis after the consumption of infected milk [18]. *M. tuberculosis* is also inactivated by heat [19]. *M. bovis* tuberculosis was indeed highly prevalent before the generalization of pasteurization [20] and it remains a public health concern in countries with non-generalized pasteurization such as sub-Saharan African countries [21] and Tunisia [22]. In Europe, deadly *M. bovis* tuberculosis is reemerging with new consumption trends such as “natural” unpasteurized dairy products [23]. Data reported here showed that *“M. canettii”* cannot grow beyond temperatures of 45–55 °C, suggesting that cooked drinks and foods are most likely not sources of contamination. Indeed, the foodstuff model based on standard methods relies on a 50 °C initial phase which may have killed the mycobacteria, but this temperature was not tested here on pure colonies. Therefore, it may be of interest to further narrow down the temperature spectrum of susceptibility by testing the colonies at 50 °C. In the context of the Republic of Djibouti, the country with the highest prevalence of *“M. canettii”* tuberculosis [5] this temperature cut-off allows to select foods that could be a potential source of infection. Contaminated drinking water supplies, recreational water and water used in food production could pose a significant risk. Cow, goat and camel milk are still consumed raw or fermented for their nutritional qualities. Also, seafood that is consumed raw such as oysters constitutes another potential source of contamination, particularly for expatriates.

### Conclusions

If drinks and food are sources of contamination by *“M. canettii”*, then the data here reported suggest that cooked foodstuffs are unlikely sources of contamination; and that water, raw milk and dairy products and seafood have to be further investigated as potential sources of contamination by *“M. canettii”*.

## Limitations

This study included 3 strains of “*M. canettii*” which may not be representative of the all spectrum of this species; especially as no environmental strain is available for such study.

## Abbreviations

OADC: oleic acid-albumin-dextrose-catalase
PBS: phosphate buffered saline
CFU: colony-forming units
